# Association of preoperative anxiety and depression symptoms with postoperative complications of cardiac surgeries*

**DOI:** 10.1590/1518-8345.2784.3107

**Published:** 2018-11-29

**Authors:** Hélen Francine Rodrigues, Rejane Kiyoma Furuya, Rosana Aparecida Spadoti Dantas, Alfredo José Rodrigues, Carina Aparecida Marosti Dessotte

**Affiliations:** 1 Confederação Nacional das Cooperativas Médicas, Hospital Unimed, Ribeirão Preto, SP, Brasil.; 2 Instituto Federal do Paraná, Campus Londrina, Londrina, PR, Brasil.; 3 Universidade de São Paulo, Escola de Enfermagem de Ribeirão Preto, Centro Colaborador da OPAS/OMS para o Desenvolvimento da Pesquisa em Enfermagem, Ribeirão Preto, SP, Brasil.; 4 Universidade de São Paulo, Faculdade de Medicina de Ribeirão Preto, Ribeirão Preto, SP, Brasil.

**Keywords:** Postoperative Care, Anxiety, Depression, Perioperative Nursing, Thoracic Surgery, Postoperative Complications

## Abstract

**Objective:**

to investigate the associations of preoperative anxiety and depression symptoms with postoperative complications and with sociodemographic and clinical characteristics of patients submitted to the first coronary artery bypass graft.

**Method:**

observational, analytical and longitudinal study. A consecutive non-probabilistic sample consisted of patients submitted to coronary artery bypass graft. To evaluate the symptoms, the Hospital Anxiety and Depression Scale was used. tracheal intubation for more than 48 hours, hemodynamic instability, sensorineural deficit, agitation, hyperglycemia, infection, nausea, vomiting, pain and death were classified as complications. The Mann-Whitney and Spearman Correlation tests were used, with a significance level of 0.05.

**Results:**

a total of 75 patients participated. The group that presented hemodynamic instability in the postoperative period had a greater median for the anxiety symptoms (p = 0.012), as well as the women (p = 0.028). The median of the depression symptoms was higher in the group presenting nausea (p = 0.002), agitation (p <0.001), tracheal intubation for more than 48 hours (p = 0.018) and sensorineural deficit (p = 0.016).

**Conclusion:**

there was association of the symptoms of preoperative anxiety with hemodynamic instability in the postoperative period and with the female gender, as well as association of depression symptoms with the following complications: nausea, agitation, time of intubation in the postoperative period and sensorineural deficit.

## Introduction

Despite the progress of science and technology in the health area, with important advances in the treatment of Cardiovascular Diseases (CVD), cardiac surgeries can cause complications that increase postoperative (PO) morbidity and mortality, even with the use of the best surgical techniques and care^(^ ^1^ ^-^ ^3^ ^)^.

The CVD set is the main cause of morbidity and mortality worldwide, mainly due to Coronary Artery Disease (CAD), which was the leading cause of death in 2013^(^ ^4^ ^)^. From the numerous clinical presentations of CVD, the focus of this study will be the coronary patients undergoing coronary artery bypass graft (CABG).

The severity of coronary patients submitted to CABG has increased considerably in the last decades, also increasing the possibility of transoperative and postoperative complications. This higher severity is related to the increased age of the patients, the presence of a greater number of women, the higher incidence of recent infarction, the greater deterioration of left ventricular function, the multiarterial involvement in the presence of a greater number of comorbidities, besides the presence in the preoperative period of renal disease and peripheral vascular disease^(^ ^5^ ^)^.

The main complications in the PO period of CABG have been addressed due to the complexity involved in the patient’s clinical condition and, consequently, in the therapeutic management. These complications may be related to the preoperative admission time; pre-existing comorbidities; inadequate living habits (e.g., smoking); advanced age; previous nutritional status (malnutrition or obesity); the type of medication used in the preoperative period; and to risk factors inherent to the anesthetic-surgical procedure^(^ ^6^ ^)^.

The indication of cardiac surgery may have a borderline character in the life of the patients, who must choose the risks inherent to the anesthetic-surgical procedure or the reduction of survival and limitation due to the symptoms of CAD^(^ ^7^ ^)^. Thus, the stress of surgical patients is unavoidable, and PO evolution may be impaired in those patients who do not develop adequate coping strategies^(^ ^8^ ^)^.

Among the many stressors experienced by patients submitted to CABG, scientific evidence was found regarding the presence of anxiety and depression symptoms^(^ ^9^ ^)^. The presence of these symptoms in the preoperative period can trigger a physiological response involving the endocrine and autonomic systems, thus influencing PO outcomes, increased risk of unwanted outcomes, as well as the length of hospital stay^(^ ^10^ ^)^.

In international studies, researchers investigated the association between preoperative anxiety and depression symptoms with PO complications, with a focus on mortality up to 30 days after surgery^(^ ^11^ ^-^ ^14^ ^)^. In the present study, we chose to investigate the presence of complications and death in the PO period, during the patients’ stay in the Intensive Care Unit (ICU). This is justified by the fact that this is the period of greatest instability and criticality, as well as due to the scarce scientific production focusing on this period.

In view of the above, the objective of the present study was to investigate possible associations between the symptoms of preoperative anxiety and depression with the presence of complications in the PO period, and also with the sociodemographic and clinical characteristics of patients submitted to the first CABG.

## Method

This is an observational and analytical study, of the longitudinal type, carried out at the inpatients hospitalization units of the Medical Clinic and Surgical Clinic of a university hospital in the interior of São Paulo state.

A consecutive and non-probabilistic sample consisted of participants who met the inclusion criteria, namely patients from both sexes, aged 18 years or older, who had undergone the first CABG, whose elective procedure was scheduled with more than 12 hours in advance, in the period from September 2013 to September 2015.

Patients who did not present cognitive conditions to answer the questionnaires or who presented clinical decompensation of heart disease on the day of data collection in the preoperative period (presence of dyspnea, precordialgia or orotracheal intubation) were excluded.

To identify the patients with cognitive conditions to answer the questionnaires, we used the instrument “Mini Mental State Examination - MMSE”^(^ ^15^ ^)^, in the adapted version to the Portuguese language^(^ ^16^ ^)^. The cut-off points adopted in this study were as follows: illiterate patients had to score at least 13 points; those with one to seven years of schooling, a minimum of 18 points; and with eight or more years of schooling, at least 26 points^(^ ^17^ ^)^.

In the preoperative period, the data were collected for the sociodemographic and clinical characterization by means of an interview with the patients and consultation of the medical charts, besides the evaluation of anxiety and depression symptoms through an interview conducted on the day before the surgery.

After a maximum of 24 hours after discharge from the ICU, the data related to the evolution of the patient in the PO period were collected by consulting the medical charts and the electronic data system used in the ICU of the said hospital. The PO period investigated in this study refers to the period of stay of the patients in the ICU, that is, from the moment of admission, after the end of the surgery, until the discharge of the unit.

For the characterization of PO complications, we analyzed the patient’s evolutions performed by the nursing and medical teams and the nursing care instrument in the PO period, which are routinely filled in the referred institution. The results of serum lactate levels and central venous oxygen saturation were obtained from the electronic data system.

For the characterization of the participants, an instrument was elaborated based on the literature review and in an earlier study^(^ ^9^ ^)^, containing sociodemographic data (birth, hospitalization and interview dates, sex, marital status, schooling, professional status and monthly family income) and clinical data (presence of associated pathologies, left ventricular ejection fraction, smoking, use of psychotropic drugs before the surgery and rescheduling of surgery). The age was calculated by subtracting the date of the interview from the date of birth, since the preoperative admission time was calculated by subtracting the date of the surgery from the admission date.

For the evaluation of left ventricular ejection fraction (LVEF), we considered values greater than or equal to 50% for preserved LVEF, and less than 50% for decreased LVEF^(^ ^18^ ^)^. These values are also used as reference in the hospital where the study was developed.

The following data were collected from the PO period in the ICU: date and time of arrival at the ICU; date and time of withdrawal of the orotracheal tube (OTT); lactate values; diuresis volume; central venous oxygen saturation; evolution of the patient in the PO period (mean arterial pressure, central venous pressure, serum creatinine levels); and date and time of discharge from the ICU. The length of stay of the OTT was calculated by subtracting the date and time of removal of the tube from the date and time of admission to the ICU. The length of stay in the ICU was calculated by subtracting the date and time of discharge from the unit from the date and time of admission.

Considering that the symptoms of preoperative anxiety and depression may act as additional stressors to patients undergoing cardiac surgeries, thus increasing the physiological response triggered by surgical trauma, the possible complications associated with these symptoms are those of a pulmonary nature (tracheal intubation under mechanical ventilation for more than 48 hours, after the surgery), cardiac (presence of hemodynamic instability), neurological (presence of sensorineural deficit and/or agitation), endocrine (presence of hyperglycemia - plasma glucose above 100mg/dL)^(^ ^19^ ^)^, infectious (diagnosis of Urinary Tract Infection, health care-related pneumonia, Bloodstream Infection and Surgical Site Infection^(^ ^20^ ^)^), digestive (presence of nausea and vomiting) and sensory nature (presence of pain) and, finally, the death occurred during the patient’s stay in the ICU.

The information related to the presence of infection in the PO period was collected, retrospectively, through the hospital infection data available at the Hospital Infection Control Commission (CCIH in Portuguese) of the said institution.

For the patient characterization, according to the hemodynamic stability (stable or unstable), the tissue perfusion markers were used. From the clinical point of view, the patient should present a mean arterial pressure (MAP) above 70 mmHg, a satisfactory level of consciousness and adequate diuresis (> 0.5 mL/kg/hour). From the biochemical point of view, patients should present serum lactate values below <2 mmol/L and central venous oxygen saturation above >70%^(^ ^21^ ^)^. The patient was classified as having “hemodynamic instability” when a concomitant alteration of three of the described parameters was observed at any time during the period of their stay in the ICU, regardless of the number of times this condition was observed.

The Hospital Anxiety and Depression Scale (HADS) instrument was used to evaluate the symptoms of preoperative anxiety and depression^(^ ^22^ ^)^, in its version adapted to Portuguese language^(^ ^23^ ^)^. The HADS instrument has 14 questions (seven for anxiety and seven for depression), which address somatic and psychological symptoms with a four-point response scale. The values of the answers range from zero to three whose sum can range from zero to 21 (twenty-one) points for each of the emotional disorders surveyed. In the present study, the evaluation of the responses was made with the total value of each subscale (HADS-anxiety and HADS-depression), and the higher the value, the greater the perception of anxiety and depression symptoms.

For the investigation of the associations of preoperative anxiety and depression symptoms with the presence of complications in the PO period of CABG during stay in the ICU (categorized orotracheal intubation, hemodynamic instability, sensorineural deficit, agitation, hyperglycemia, presence of infection, nausea, vomiting, and pain), and the sociodemographic characteristics (gender, age, marital status and professional status), the non-parametric Mann-Whitney statistical test was used for two independent samples. The hypothesis test was performed when at least four patients presented the complication.

In order to investigate the correlation of preoperative anxiety and depression symptoms with clinical characteristics (preoperative admission time, surgery time and length of stay in the ICU), the non-parametric Spearman Correlation test was used. For the analysis of the strengths of linear correlation between the measurements, we used the classification that considers values lower than 0.30, even when statistically significant, without clinical relevance; values between 0.30-0.50 indicate moderate correlation and above 0.50, strong correlation^(^ ^24^ ^)^. The level of significance was set at 0.05.

The research project was prepared in accordance with the ethical precepts of Resolution 466/2012 and approved by the Research Ethics Committee of the School of Nursing of Ribeirão Preto, University of São Paulo, under Official Letter No. 191/2013. Patients were invited to participate in the study and, after agreeing, we read the Informed Consent Form (ICF), which was signed by the patient and the main investigator in two copies, of which one was handed to the patient and the other was archived by the researcher.

## Results

During the data collection period, from September 2013 to September 2015, 296 cardiac surgeries were performed. Of this total, 75 patients met the inclusion criteria and accepted to participate in the study.

Most of the patients were male (n = 52, 69.3%), married or living in a stable union (n = 53, 70.7%) and did not perform paid activity before surgery (n = 50, 66.7%).

The mean age of the patients was 61.8 years (SD = 9.8), ranging from 40 to 83 years. The mean number of years of study was 5.4 (SD = 4.3) and the average monthly family income (in reais) was R$ 2,687.6 (SD = 2,903.7).

The preoperative clinical characterization of patients is shown in Table 1.

**Table 1:** – Preoperative characterization of the 75 patients submitted to coronary artery bypass graft according to the manifestation of coronary artery disease, the presence of comorbidities, life habits and the use of psychotropic drugs. Ribeirão Preto, SP, Brazil, 2013-2015

Variable	n (%)
Manifestation of CAD*	
Recent AMI^†^	21 (28.0)
Previous AMI^†^	20 (26.7)
Stable angina	13 (17.3)
Unstable angina	9 (12.0)
Not described in medical charts	12 (16.0)
Comorbidities	
Systemic Arterial Hypertension	63 (84.0)
Overweight/Obesity	56 (74.6)
Diabetes mellitus	52 (69.3)
Dyslipidemia	45 (60.0)
Hypothyroidism	10 (13.3)
Chronic Renal Insufficiency	9 (12.0)
Cardiac insufficiency	8 (10.7)
Chronic obstructive pulmonary disease	3 (4.0)
Life habits	
Previous smoking	32 (42.7)
Current smoking	13 (17.3)
Preoperative psychotropic use	
Yes	16 (21.3)

*CAD: Coronary artery disease; †AMI: Acute myocardial infarction

Regarding the left ventricular ejection fraction (LVEF) of the preoperative patients, 47 (62.7%) had preserved LVEF, while 28 (37.3%) presented reduced LVEF.

The surgical procedure was rescheduled for 27 (36.0%) patients, being canceled once for 21 (28.0%) subjects; twice for five (6.7%) subjects; and three times for one (1.3%) subject.

The mean preoperative time was 14.5 days (SD = 10.0, median = 13.0), ranging from one day to 42 days.

The characterization of postoperative complications in patients undergoing CABG is shown in Table 2.

**Table 2:** – Characterization of the postoperative period of the 75 patients submitted to coronary artery bypass graft according to the presence of endocrine, sensory, cardiac, digestive, neurological, pulmonary, and infectious complications and death. Ribeirão Preto, SP, Brazil, 2013-2015

Variable		n (%)
Presence of Complications		
Endocrine	Hyperglycemia	73 (97.3)
Sensory	Pain	66 (88.0)
Cardiac	Hemodynamic instability	47 (62.7)
Digestive	Nausea	41 (54.7)
	Vomiting	18 (24.0)
Neurological	Agitation	13 (17.3)
	Sensorineural Deficit	4 (5.3)
Pulmonary	OTT* for more than 48 hours	5 (6.7)
Infeccious	Organ or cavity SSI^†^	2 (2.7)
	Deep incisional SSI^†^	1 (1.3)
	Pneumonia^‡^	1 (1.3)
	Bloodstream infection	1 (1.3)
Death	Yes	1 (1.3)

*OTT: Orotraqueal tube; †SSI: Surgical Site Infection; ‡Pneumonia: healthcare-associated pneumonia

The mean length of stay in the ICU was 3.5 days (SD = 2.3, median = 3.0), ranging from 2 to 18 days.

Regarding the preoperative symptoms of anxiety and depression, the studied patients presented a mean of 5.5 (SD = 4.6, median of 4.0) for the anxiety symptoms, ranging from zero to 17. For the depression symptoms, the mean values were 4.0 (SD = 3.9, median 3.0), ranging from zero to 18.

Table 3 shows the medians and values, minimum and maximum, obtained from anxiety and depression symptoms, according to the presence of complications in the PO period of the patients submitted to the CABG.

**Table 3:** – Descriptive analysis of the anxiety and depression symptoms of the 75 patients who had undergone coronary artery bypass graft according to the presence of postoperative complications and probability (*p*) values associated with the Mann-Whitney test. Ribeirão Preto, SP, Brazil, 2013-2015

Variable	HADS* - Anxiety		HADS* - Depression	

	Median	Minimum - maximum	Median	Minimum - maximum
Hyperglycemia				
Yes (n = 73)	4.0	0 – 17	3.0	0 – 18
No (n = 2)	5.5	1 – 10	5.5	0 – 11
*p* ^†^	0.987		0.947	
Pain				
Yes (n = 66)	4.0	0 – 17	3.0	0 – 18
No (n = 9)	4.0	0 – 8	1.0	0 – 11
*p* ^†^	0.252		0.155	
Hemodynamic Instability				
Yes (n = 47)	5.0	0 – 16	3.0	0 – 18
No (n = 28)	3.0	0 – 17	3.0	0 – 14
*p* ^†^	0.012^‡^		0.778	
Nausea				
Yes (n = 41)	5.0	0 – 17	4.0	0 – 18
No (n = 34)	3.5	0 – 16	2.0	0 – 11
*p* ^†^	0.068		0.002^‡^	
Vomiting				
Yes (n = 18)	5.0	0 – 16	3.0	0 – 11
No (n = 57)	4.0	0 – 17	3.0	0 – 18
*p* ^†^	0.353		0.915	
Agitation				
Yes (n = 13)	5.0	0 – 17	7.0	0 – 14
No (n = 62)	4.0	0 – 16	2.0	0 – 18
*p* ^†^	0.343		<0.001^‡^	
OTT^§^ for more than 48 hours				
Yes (n = 5)	12.0	4 – 15	10	3 – 11
No (n = 70)	4.0	0 – 17	3	0 – 18
*p* ^†^	0.067		0.018^‡^	
Sensorineural Deficit				
Yes (n = 4)	9.5	4 – 15	10.5	4 – 11
No (n = 71)	4.0	0 – 17	3.0	0 – 18
*p* ^†^	0.118		0.016^‡^	

*HADS: Hospital Anxiety and Depression Scale; †*p*= p-value from the Mann-Whitney test; ‡*p* <0.05: statistical significance; §OTT: Orotraqueal Tube

Table 4 presents the medians and the minimum and maximum values of anxiety and depression symptoms according to sex, age, marital status and employment status of the 75 patients who had undergone CABG.

**Table 4:** – Descriptive analysis of the anxiety and depression symptoms of the 75 patients who had undergone coronary artery bypass graft according to sex, age, marital status, employment status, and probability (*p*) values associated with the Mann-Whitney test. Ribeirão Preto, SP, Brazil, 2013-2015

Variable	HADS* - Anxiety		HADS* - Depression	

	Median	Minimum - maximum	Median	Minimum - maximum
Sex				
Male (n = 52)	4.0	0 – 16	3.0	0 – 11
Female (n = 23)	6.0	0 – 17	3.0	0 – 18
*p* ^†^	0.028^‡^		0.109	
Age				
Elderly (n = 46)	4.0	0 – 16	3.0	0 – 18
Adult (n = 29)	4.0	0 – 17	2.0	0 – 14
*p* ^†^	0.965		0.507	
Marital status				
With partner (n = 53)	4.0	0 – 16	3.0	0 – 18
Without partner (n = 22)	5.0	0 – 17	3.0	0 – 14
*p* ^†^	0.365		0.422	
Employment relationship				
Inactive (n = 50)	5.0	0 – 17	3.0	0 – 18
Active (n = 25)	4.0	0 – 15	3.0	0 – 11
*p* ^†^	0.379		0.455	

*HADS: Hospital Anxiety and Depression Scale; †*p*= p-value from the Mann-Whitney test; ‡*p* <0.05: statistical significance

A statistically significant difference was found only in the evaluation of anxiety, whose values were higher for the female than for the male (*p* = 0.028).

The measures obtained for the depression symptoms were similar when comparing patients according to age, marital status and employment relationship.

The results of the correlations between the anxiety and depression symptoms and the clinical characteristics (preoperative admission time, surgery time and length of stay in the ICU) are presented in Table 5.

**Table 5:** – Correlation of anxiety and depression symptoms with the clinical characteristics of the 75 patients who had undergone coronary artery bypass graft with the respective probability values (*p*) associated with the Spearman correlation test. Ribeirão Preto, SP, Brazil, 2013-2015

Variable	HADS* - Anxiety		HADS* - Depression	

Clinical features	r^†^	p^‡^	r^†^	p^‡^
Preoperative admission time (days)	0.120	0.303	0.004	0.972
Surgery time (minutes)	0.076	0.517	0.008	0.943
Length of stay in the ICU^§^ (days)	0.100	0.933	0.178	0.127

*HADS: Hospital Anxiety and Depression Scale; †r= correlation strength from the Spearman correlation test; ‡*p*= statistical significance of the correlation from the Spearman correlation test; §ICU: Intensive Care Unit

The correlations found between anxiety and depression symptoms with preoperative admission time, surgery time and length of stay in the ICUwere weak, less than 0.30 and did not present statistical significance.

## Discussion

The hypothesis of this study was that patients with more anxiety and depression symptoms in the preoperative period would present a higher frequency of complications in the PO period of CABG during their stay in the ICU. This hypothesis was partially confirmed. Concerning anxiety, it was observed that patients who had more anxiety symptoms showed higher frequency of hemodynamic instability in the PO period than those with fewer symptoms. Regarding the symptoms of preoperative depression, it was verified that the patients who had more symptoms of depression had more frequent nausea, agitation, presence of OTT for more than 48 hours and neurological deficit in the PO period, during the stay in the ICU, than those with fewer symptoms.

Given the originality of the present research, it is not possible to compare these results with those that could be obtained in other groups of patients undergoing CABG or other cardiac surgeries. Some studies have investigated the relationship of preoperative anxiety and depression symptoms of patients undergoing cardiac surgeries with complications and PO mortality, but the PO period varied widely among the studies, in addition to the lack of consensus on the best or more indicated tool to evaluate the symptoms of anxiety and depression^(^ ^11^ ^-^ ^14^ ^)^.

The occurrence of death in the postoperative period, during the patients’ stay in the ICU, among patients submitted to CABG, was 1.3%. As the patient was not followed throughout the whole PO period and patients who died in the operating room were not included, the results of this study cannot be compared with mortality rates found in the international and national literature.

In any case, this is a very relevant topic because, in clinical practice, patients’ emotional symptoms are poorly evaluated, especially when considering the severity of the cardiovascular disease and the presence of numerous patient comorbidities in the perioperative period of these surgeries.

An international study was carried out in Australia with 158 patients undergoing CABG, with or without concomitant valve procedures, in which the authors showed that generalized anxiety in the preoperative period was a significant predictor for the occurrence of cardiovascular and cerebrovascular complications in the PO period^(^ ^14^ ^)^.

In a study conducted in Germany, the authors evaluated the efficacy of a short-term, information-based intervention and emotional support to reduce preoperative anxiety and in the PO period of patients undergoing CABG. They found that the group of patients who received the intervention reported a moderately lower state of anxiety after the intervention and prior to surgery, as well as in the PO period, five days after surgery, with statistically significant differences between groups (p <0.001); however, it did not show a significant difference in mortality in the PO period and in the length of stay in the ICU^(^ ^11^ ^)^.

In Sweden, researchers retrospectively evaluated 56,064 patients who had undergone CABG between 1997 and 2008. The objective was to determine the possible association of preoperative depression symptoms with long-term survival, that is, until the occurrence of death from any cause or readmission due to myocardial infarction, heart failure or stroke. They found that 324 (0.6%) patients had preoperative depression. During an average follow-up of 7.5 years, 35% of these patients with depression evolved to death, compared to 25% in the non-depressed group. They concluded that preoperative depression was significantly associated with increased long-term mortality^(^ ^13^ ^)^.

Researchers from a major American study concluded that depression is an important predictor of mortality following cardiac surgeries, regardless of risk factors, such as smoking, age, sex, diabetes mellitus, prior myocardial infarction, number of grafts and LVEF, since, after adjustment for these factors, depression was associated with a two- to three-fold increase in mortality risk^(^ ^25^ ^)^. Depression was also pointed out in other studies as an independent risk factor for mortality after CABG^(^ ^26^ ^-^ ^27^ ^)^.

The present study also proposed to evaluate the association of preoperative anxiety and depression symptoms with the sociodemographic characteristics (sex, age, marital status and professional status) of the patients. It was found that women had the highest median for anxiety symptoms when compared with men, and the difference found was statistically significant, that is, women had more preoperative anxiety symptoms than men.

The reasons that may explain the increased frequency of anxiety symptoms among women are still unknown and need to be further investigated. However, some hypotheses for this scenario have been raised. Among the factors that could contribute to the observed differences between men and women are the biological factors, such as the influence exerted by female sex hormones and psychosocial factors, such as the overload of women’s roles with the recent changes in society^(^ ^28^ ^)^.

Other authors have shown that women were more convinced of their illnesses, while men denied the stress associated with surgery more frequently^(^ ^29^ ^)^. Thus, another possible reason for the higher frequency of these symptoms among women would be greater ease in expressing their feelings. Corroborating this idea, in another study, the authors describe that men are more likely to report symptoms of anger, irritability, risk behaviors and substance abuse, rather than more traditional symptoms of anxiety and depression, such as sleep disorders and social isolation^(^ ^30^ ^)^.

In the present study, men and women presented the same median value for the depression symptoms, a result that differs from the literature. In the national and international literature, the relationship between female sex and the symptoms of preoperative depression has been established, with several studies showing women with more depression symptoms in the preoperative period of CABG^(^ ^12^ ^-^ ^13^ ^,^ ^31^ ^-^ ^32^ ^)^, being the female sex a risk factor for the development of depression before the CABG^(^ ^31^ ^)^. These differences may result from the application of several instruments for the evaluation of the symptoms, as well as the heterogeneity of the moments in which the data were collected, that is, with which anticipation from the surgery the symptoms were evaluated.

In this study, no differences were found in the evaluation of anxiety and depression symptoms in relation to age, marital status and employment status. However, a higher frequency of anxiety and depression symptoms was previously documented among younger patients undergoing CABG^(^ ^27^ ^,^ ^32^ ^)^. It has also been identified that patients undergoing CABG who are supported by their families, especially their spouses, recover more quickly than those who do not have such support^(^ ^33^ ^)^, thus evidencing the impact of the marital status on the coping of the patient submitted to the surgery. In the same way, it is already described in the literature that the preoccupation with work or with being away from it is a factor that bothers the patients in the preoperative period of CABG^(^ ^34^ ^)^.

Regarding the correlations between preoperative anxiety and depression symptoms and preoperative admission time, surgery time and length of stay in the ICU in the subjects submitted to CABG, the correlations found in this study were weak and without statistical significance. In a study carried out in Greece, the authors also did not find a relation between the symptoms of preoperative depression and the preoperative admission time, corroborating with the results of this research^(^ ^32^ ^)^. On the other hand, in a study developed in Spain, the authors evidenced that the preoperative admission time greater than three days was a determining factor for the onset of depression symptoms, before the CABG^(^ ^27^ ^)^. Regarding the surgery time and the length of stay in the ICU, no studies were found in the literature correlating these data with the symptoms of preoperative anxiety and depression.

One of the limitations of the study was the fact that all participants were cared for in a single public health institution. We did not investigate patients with a higher socioeconomic status, who were treated in private hospitals, or even patients treated in different health services linked to the Unified Health System. The second limitation refers to the way data on most PO complications were collected, that is, through the collection of information in the patient’s chart. In order to identify the complications during ICU admission, this information had to be described in the medical chart.

The results of the present study are relevant to clinical practice because although the instrument used for symptom investigation does not provide the diagnosis of mood disorder, it may be useful to identify patients who present anxiety and/or depression symptoms and thus allow the access of these patients to pharmacological or psychotherapeutic interventions, making the perioperative period healthier, with a view to PO recovery.

The investigation of anxiety and depression symptoms should be part of routine preoperative assessment of nurses. Therefore, it is necessary that nurses understand these disorders by recognizing their relevance to the evolution in the PO period. It is necessary to establish with the patient a relationship of trust, which allows the patient to expose their afflictions, fears, worries and anxieties, as well as to acquire information that they deem necessary on the surgical process they are going through. In this scenario, nurses have an important role, since they are the main responsible for nursing guidance throughout the perioperative period.

Preoperative education, in addition to emphasizing and addressing risk factors and the ways of minimizing them, must meet the personal demands of each patient. It is known that the hospital environment, the care routines and the times of each process can be extremely strange to the patient and can cause symptoms of anxiety and depression. However, much more than inferring what patients need to know, it becomes necessary to know the particular needs of each one in order to address each one in an equitable manner. After all, caring for the patient in their entirety, focusing not on the disease, but on the individual, is essential for quality care.

## Conclusion

In the present study, we found an association of anxiety symptoms in the preoperative period with the hemodynamic instability of patients in the PO period and with the female sex. It was also identified association of depression symptoms in the preoperative period with nausea, agitation, neurological deficit and length of stay of the OTT in the PO period, during the stay in the ICU.
